# Comparing Two Neurodevelopmental Disorders Linked to CK2: Okur-Chung Neurodevelopmental Syndrome and Poirier-Bienvenu Neurodevelopmental Syndrome—Two Sides of the Same Coin?

**DOI:** 10.3389/fmolb.2022.850559

**Published:** 2022-05-26

**Authors:** Demetra Ballardin, Jose M. Cruz-Gamero, Thierry Bienvenu, Heike Rebholz

**Affiliations:** ^1^ INSERM U1266, Institute of Psychiatry and Neuroscience of Paris, Université de Paris, Paris, France; ^2^ GHU-Paris Psychiatrie et Neurosciences, Hôpital Sainte Anne, Paris, France; ^3^ Service de Médecine Génomique des Maladies de Système et d’organe, Hôpital Cochin, APHP, Centre Université de Paris, Paris, France; ^4^ Center of Neurodegeneration, Faculty of Medicine, Danube Private University, Krems, Austria

**Keywords:** OCNDS, POBINDS, NDD-neurodevelopmental disorder, CK2 (casein kinase II), autism-spectrum disorders (ASD)

## Abstract

In recent years, variants in the catalytic and regulatory subunits of the kinase CK2 have been found to underlie two different, yet symptomatically overlapping neurodevelopmental disorders, termed Okur-Chung neurodevelopmental syndrome (OCNDS) and Poirier-Bienvenu neurodevelopmental syndrome (POBINDS). Both conditions are predominantly caused by *de novo* missense or nonsense mono-allelic variants. They are characterized by a generalized developmental delay, intellectual disability, behavioral problems (hyperactivity, repetitive movements and social interaction deficits), hypotonia, motricity and verbalization deficits. One of the main features of POBINDS is epilepsies, which are present with much lower prevalence in patients with OCNDS. While a role for CK2 in brain functioning and development is well acknowledged, these findings for the first time clearly link CK2 to defined brain disorders. Our review will bring together patient data for both syndromes, aiming to link symptoms with genotypes, and to rationalize the symptoms through known cellular functions of CK2 that have been identified in preclinical and biochemical contexts. We will also compare the symptomatology and elaborate the specificities that distinguish the two syndromes.

## Introduction

Nearly 15% of children in industrialized countries are affected by neurodevelopmental disorders (NDDs) as estimated by the World Health Organization (World Health Organization, 2013). NDDs are a group of conditions characterized by delayed or impaired functions and maturation of the central nervous system, including disorders such as autism spectrum disorder (ASD), intellectual disability (ID) and learning disorders (LD) (Gilissen et al., 2014). Even though some cases have been linked to environmental exposures (Dietrich et al., 2005), most NDDs likely result from the combination of genetic and environmental risk factors; and the role of genetics, especially of single-gene variants, has gathered the attention (Soden et al., 2014). Until recently, diagnoses have been mainly phenotype-driven, however, enhanced use of sequencing technologies such as targeted gene panels, whole-exome (WES) and whole-genome sequencing (WGS) have enabled an unbiased genotype-driven diagnosis (Aronson and Rehm, 2015; Fitzgerald et al., 2015).

WES analysis linked several *de novo* variants in the gene *CSNK2A1*, located on chromosome 20 (20p13), which encodes for the catalytic subunit of CK2 (CK2α) to a novel neurodevelopmental syndrome, now termed Okur-Chung Neurodevelopmental Syndrome (OCNDS) (Okur et al., 2016; OMIM #617062), characterized by developmental delay, intellectual disability, hypotonia, behavioral problems (social interaction deficits, hyperactivity, and repetitive movements), language/verbalization deficits, and, in some cases, epilepsy (Trinh et al., 2017; Akahira-Azuma et al., 2018; Chiu et al., 2018; Colavito et al., 2018; Owen et al., 2018; Nakashima et al., 2019; Martinez-Monseny et al., 2020; Xu et al., 2020; Wu et al., 2021). To date, 35 cases are described in the literature. Shortly after the first report of OCNDS, Poirier et al. linked variants of the regulatory subunit of CK2 (*CSNK2B*) to a neurodevelopmental disorder characterized by early-onset seizures, mainly generalized tonic-clonic seizures (GTCS), and ID, growth retardation and other clinical features (Poirier et al., 2017; OMIM #618732). This syndrome, linked to the *CSNK2B* gene located on chromosome 6 (6p21.33), now termed the Poirier-Bienvenu Neurodevelopmental Syndrome (POBINDS), has been described in 51 patients (Sakaguchi et al., 2017; Li et al., 2019; Nakashima et al., 2019; Bonanni et al., 2021; Ernst et al., 2021; Wilke et al., 2022).

Casein kinase 2 (CK2) is a ubiquitous, highly conserved and constitutively active serine/threonine protein kinase which can utilize ATP or GTP as phosphate donor (Niefind et al., 1999). In eukaryotic cells, CK2 is a tetrameric complex composed of two α and/or α’ and two β subunits, all of which are encoded by different genes (Niefind et al., 2001). In the brain, CK2α is more abundant than in other tissues, with a predominance of the α subunit over α’ (Ceglia et al., 2011). CK2 is localized in different cellular compartments, is involved in diverse processes such signal transduction, replication, translation, and metabolism (Roffey and Litchfield, 2021), as well as roles in angiogenesis (Montenarh, 2014), development and differentiation (Götz and Montenarh, 2017), and the immune system (Hong and Benveniste, 2021). Moreover, it is upregulated in many cancers (Ahmad et al., 2005; Ruzzene and Pinna, 2010; Rowse et al., 2017). Interestingly, CK2 has been implicated in SARS-Cov2 infection since mass spectrometric analysis revealed an upregulation of CK2 mediated phosphorylation events in response to virus infection in Vero E6 kidney cells (Bouhaddou et al., 2020).

Various mouse models with altered CK2 expression attest to the indispensability of this kinase in mammalian brain development and function: CK2α^−/−^ mice are not viable and die at E11.5 due to heart and brain maldevelopment, while CK2α^+/−^ mice did not show any overt gross phenotype (Lou et al., 2008; Seldin et al., 2008). Mice with a conditional CK2α KO in dopamine D1 receptor (D1R) expressing neurons, exhibit hyperlocomotion and motor deficiencies which were linked to elevated D1R activity (Rebholz et al., 2013). Loss of CK2β has an even more deleterious effect on survival since CK2β^−/−^ embryos are absorbed very early during embryogenesis, at E7.5 (Buchou et al., 2003). Heterozygous CK2β mice are generally healthy and reproductive, however they are born at a lower-than-expected ratio, with a 30% reduction of heterozygous live offspring and 20% of live mice exhibiting stunted growth and malformations (Blond et al., 2005).

## Comparison of Symptom Profile

Being classified as NDDs, it is not surprising that both, OCNDS and POBINDS, share many phenotypic similarities with other NDDs and between themselves. Indeed, around 80% of OCNDS and POBINDS patients present growth deficits, in terms of microcephaly, stature and weight, and a high prevalence of dysmorphic features (Table 1). In both syndromes, this is accompanied by developmental delay in terms of motor and speech milestones; however, while POBINDS patients achieve walking and talking on average around the second year of life (22.5 and 24.5 months, respectively), these milestones are more delayed in OCNDS patients, with walking achieved on average at 27.6 months and talking at 42.9 months (Table 1). Moreover, intellectual disability seems to be more prominent in OCNDS compared to POBINDS patients, with 94 and 85% of patients affected, respectively. Patients from both syndromes present neurological and behavioral problems with similar prevalence: hypotonia (77% for both OCDNS and POBINDS) and autistic features (55 and 56%, for OCDNS and POBINDs, respectively); hyperactivity (17% compared to 13% of POBINDS), or ADHD-like features (38 and 44%). 58% of OCNDS patients present stereotyped movements, a phenotype that was not noticed in POBINDS patients (based on seven cases where this symptom was specifically addressed). The most striking phenotypic difference clearly is epilepsy. While only 38% of OCNDS patients suffer from seizures, mainly absences or febrile types, 90% of POBINDS patients present epilepsy and 42% of those suffer from generalized tonic-clonic seizures (GTCS). This correlates well with 60% of POBINDS patients having an abnormal EEG, while MRI anomalies were more prominent in OCNDS patients (52% compared to 37%). Notably, 31% of OCDNS patients of whom MRI was undertaken, exhibit anomalies in the pituitary gland. Interestingly, 77 and 58% of OCNDS patients were reported having sleeping and gastrointestinal problems (i.e., feeding difficulties, constipation), respectively, which were not reported by parents of POBINDS patients.

**TABLE 1:** Comparison of OCNDS and POBINDS: types of variants and symptoms.

		OCNDS		POBINDS	
		Cases	%	Cases	%
Mutation	Missense	32/35	91	20/48	41
	Nonsense	0/35	0	8/48	17
	In-frame duplication	0/35	0	1/48	2
	Frameshift	0/35	0	7/48	15
	Start loss	1/35	3	3/48	6
	Splice site	2/35	6	9/48	19
Growth	Microcephaly	8/10	80	12/14	86
	Short stature	27/31	87	21/23	91
	Underweight	23/27	85	11/13	85
	Delayed bone age	4/8	50	1/3	*
Development	Walking onset	Average 27.6 mo (based on 27 cases)		Average 22.5 mo (based on 24 cases)	
	Speech onset	Average 42.9 mo (based on 14 cases)		Average 24.5 mo (based on 12 cases)	
—	Dysmorphic features	18/21	86	18/20	90
—	ID	15/16	94	34/40	85
Neurological or behavioural problems	Hypotonia	17/22	77	15/20	75
	Autistic features	6/11	55	9/16	56
	Stereotyped behaviour	7/12	58	0/7	0
	Hyperactivity	1/6	17	1/8	13
	ADHD features	3/8	38	4/9	44
	Seizures	8/22	36	41/45	91
	Seizures: GTCS	0/8	0	18/41	44
Problems	Sleep	10/13	77	1/1	*
	Eating/gastro	14/24	58	5/5	*
	Muscolo-skeletal	5/5	*	3/3	*
Anomalies	EEG	1/3	*	12/20	60
	MRI	12/23	52	10/25	40
	Pituiary gland	4/13	31	—	—

For OCNDS, 35 and for POBINDS, 48 patient profiles were published. The respective publications are referenced in Tables 2A, 2B, Supplementary Tables S1, S2. The percentages of occurring symptoms were calculated as number of cases with symptoms divided by the number of cases where the particular symptom was tested. Therefore, we decided to not calculate the incidence ratio if a symptom was assessed in five or less patients, since this clearly would have skewed the ratio towards a misleading high percentage. These cases are marked with *. Abbreviations: mo: months; ID: intellectual disability; ADHD: attention deficit hyperactivity disorder; GTCS: generalized tonic-clonic seizure; EEG: electroencephalography; MRI: magnetic resonance imaging.

## Variants in Functional Domains

### Okur-Chung Neurodevelopmental Syndrome

The CK2α protein consists of 391 amino acids (AA) and contains five main regions of interest, all located within the kinase domain: the N-terminal domain (AA 1–38) (Niefind et al., 1998, 2001; Sarno et al., 2002), the ATP/GTP binding loop (AA 45–53) (Jakobi and Traugh, 1992; Niefind et al., 1998), basic cluster (AA 68–80) (Sarno et al., 1996; Niefind et al., 1998), active site (D156) (Niefind et al., 1998), and activation segment (AA 175–201) (Niefind et al., 1998) (Supplementary Table S1).

OCDNS-linked *CSNK2A1* variants can be found along the whole amino acid sequence and along the whole kinase domain with the exception of β4/β5 region that is located between the basic cluster and the active site, and the extreme C-terminus. The lack of variants in these regions could either indicate that the variants have a more deleterious effect on protein function or more probably have no impact. Indeed, variants in the C-terminus exist, but were designated as benign or of uncertain significance (GnomAD.broadinstitute.org). Furthermore, a C-terminally deleted CK2α protein is fully functional *in vitro* (Olsen et al., 2008). For almost all patients, variants are *de novo* missense and mono-allelic. One case of a patient with a full *CSNK2A1* gene deletion is known (https://www.sfari.org). The N-terminal domain (AA 1–38) of CK2α closely interacts with the activation segment and contributes to its activity (Niefind et al., 1998; Niefind et al., 2001; Sarno et al., 2002). One patient has a missense variant p.(E27K) in this domain. Eight children carry variants in the ATP/GTP binding domain: three patients with p.(R47Q), three patients with variants at AA position 50 [p.(Y50C) or p.(Y50S)] and two at AA position 51 [p.(S51R) or p.(S51N)]. All variants in this domain were associated with delays in growth, motor and speech development and, in general, patients had dysmorphic features and hypotonia. A short basic cluster (AA 74–80) interacts with the N-terminal region and the activation segment and is known as the substrate recognition site (Niefind et al., 1998). Variants in two positions in three patients, in or adjacent to this region, have been found at positions 73 and 80 [p.(V73E), p.(R80C), p.(R80H)] leading to different degrees of symptom severity. Interestingly, one patient exhibits a variant in the active site, p.(D156H). He has microcephaly and brachycephaly, and delayed motor development and ID. The activation segment (AA 175–201) contains the basic “p + 1 loop” that helps to recognize acidic residues of the substrate. Two patients carry the p.(D175G) variant in the “p + 1 loop”. Despite an identical amino acid change, the symptomatology and severity differ between the two patients (Table 2A and Supplementary Table S1). 12 other patients had a variant in the “p + 1 loop” of which the p.(K198R) is the most commonly described thus far (nine patients). Maybe these patients exemplify best the variability in terms of symptoms: while they share abnormalities such as delayed growth, motor and speech development, and dysmorphic features, only four had ID, six had hypotonia, three experienced seizures, one sleep apnea and one needed a G-tube and had severe gastroesophageal reflux disease (GERD). Such clinical heterogeneity clearly highlights the complexity of genotype–phenotype correlations and may point towards unknown additional modifiers that are either environmental or genetic, such as expression level variations of the different isoforms. Indeed, five different *CSNK2A1* isoforms have been identified: NM_001895.4 (13 exons, 12 coding and one uncoding exon), NM_177559.3 (12 exons with initiation codon in exon 5) that could lead to a difference of phenotype between patients carrying variants before or after exon 5, NM_0013627770.2 (14 exons with an additional exon 15), NM_001362771.2 (15 exons with an additional uncoding exon 2 and uncoding exon 15), and NM_177560.3 (14 exons with an additional uncoding exon 2). Two pathogenic variants also exist at the C-terminal end of the kinase domain: R312Q and R312W. When proteins carrying these variants are expressed in mammalian cells, they express a punctuate pattern within the cytosol, unlike other OCNDS-linked CK2α mutants and wild type CK2α. Furthermore, they are expressed at significantly lower levels than wild type CK2α (Dominguez et al., 2021). Another variant that is expressed at lower protein levels compared to wild type is the CK2α p.(R47Q), while the p.(R47G) (patient not published) expresses normally (Dominguez et al., 2021). These findings of altered expression and localization indicate that the heterogeneity of the condition may in part be caused by different variants, which may trigger different cellular responses that may participate in disease etiology. However, as described above, even when patients share the exact same variants, there is clinical heterogeneity. The C-terminus of CK2α, starting at AA 351, is phosphorylated in a cell cycle-dependent manner by Cdk1 (St-Denis et al., 2009), however, no mutants were described thus far in this domain.

**TABLE 2A:** OCNDS patients and their symptom profiles.

	Structural domain			**Kinase domain**																	
		**N-Term**									**Active site**				**p+1 loop**				C-term			
				**ATP/GTP binding domain**					**Basic cluster**					**Activation segment**								
	Variant p.(...)	M1?	E27K	R47Q	Y50S	Y50C	S51N	S51R	V73E	R80H	D156H	H160R	I174M	D175G	R191Q	R191*	F197I	K198R	P231R	R312W	R312Q	Splice site variants
	Growth delay	-	+	+/?	+	+	+	+	+	+	+	+	+	+	+	+	+	+/-	+	+	+	+/-/?
Developmental delay	Motor: walking onset (mo)	?	22	+	20	+	24	30	+	+	22	18	30	+/?	18	18	24	+/?	+	27	20	+
	Speech onset (mo)	?	36	+	12	+	36	+	+	+	+	12	18	+/?	36	+	48	+/?	+	14	54	?
Dysmorphic features		?	+	+/?	+	+/?	?	+	?	+/?	+	+	?	+	?	-	?	+/?	+	?	+	-
ID/LD		+	?	+/?	+	+/?	?	?	+	?	+	+	?	+/?	?	?	?	+/-/?	?	?	+	+/?
Neurological and behavioural problems	Hypotonia	+	?	+/-	+	?	-	+	?	+/?	?	?	?	+/?	?	+	-	+/-/?	+	+	?	+/?
	Autistic features	+	+	-/?	?	?	-	?	?	+/?	?	?	+	?	?	?	-	+/-/?	?	+	?	?
	Stereotyped behaviour	?	+	-/?	?	+/?	-	?	?	?	+	?	+	+/?	?	?	-	+/-/?	?	?	?	+/?
	Hyperactivity	?	?	-/?	?	?	-	?	?	?	?	?	?	?	?	?	-	+/-/?	?	?	?	?
	ADHD features	?	?	+/-/?	?	?	-	+	?	?	?	?	?	?	?	?	-	-/?	?	?	?	+/?
	Seizures	?	?	-/?	+	?	-	+	?	-/?	-	+	-	+/?	-	+	-	+/-/?	?	-	?	-/?
Problems	Sleep	+	+	-/?	-	-/?	?	+	+	?	+	?	?	+	?	?	?	+/-/?	?	?	?	+
	Eating/gastro intestinal	+	+	+/-	+	+/?	-	+	+	-/?	-	?	+	+	+	?	-	+/-/?	?	-	?	+/?
Anomalies	EEG	?	?	?	-	?	?	?	?	?	?	+	?	?	?	?	?	?	?	?	?	-/?
	MRI	-		+/-	-	+/?	?	?	-	+/-	+	+	?	+/?	-	-	+	+/-/?	-	?	?	-

All patients whose symptoms were published thus far are ordered by the position of their amino acid alteration, from left to right: N-terminus to the C-terminus. Patients with the same variant are grouped in one column. “+” indicates that symptom is present, “−” that it is absent, “+/−” is used when patients were described who had or had not the specific phenotype, “?” is used when the specific phenotype was not discussed in the corresponding publication. When possible, age indications (in months) for symptom onset/detection are given. Abbreviations: ATP: adenosine triphosphate; GTP: guanosine triphosphate; ID: intellectual disability; LD: learning disability; ADHD: attention deficit hyperactivity disorder; GTCS: generalized tonic-clonic seizure; GERD: gastroesophageal reflux disease; G-tube: Gastrostomy tube; EEG: electroencephalography; MRI: Magnetic resonance imaging. For more details, Supplementary Table S1. Information derived from (Trinh et al., 2017; Akahira-Azuma et al., 2018; Chiu et al., 2018; Colavito et al., 2018; Owen et al., 2018; Nakashima et al., 2019; Martinez-Monseny et al., 2020; Xu et al., 2020; Wu et al., 2021).

### Poirier-Bienvenu Neurodevelopmental Syndrome

POBINDS-linked variants in *CSNK2B* can be found along the exonic as well as intronic sequences, and no clear variant hotspots can be identified. While for CK2α the predominant type of variant was missense (91%), for CK2β, various variant types are present: eight splicing site variants were thus far identified, compared to only two in OCNDS. Generally, such variants may lead to retention of large segments of intronic DNA, or to entire exons being spliced out of the mRNA, resulting in the production of a nonfunctional protein. Three POBINDS and one OCNDS patients have start loss variants, resulting either in loss of the protein entirely or a N-terminally truncated protein if an alternative start codon is used. Interestingly, while two of these patients suffered from seizures, none of them suffered from GTCS.

The CK2β protein consists of 215 AA and contains three major domains: the Asp/Glu-rich acidic domain (AA 54–64) (Li et al., 1996; Litchfield et al., 1996), metal binding (zinc finger domain) (AA 105–146) (Chantalat, 1999), and C-terminal alpha subunit interaction domain (AA 171-end) (Marin et al., 1997; Chantalat, 1999; Sarno et al., 1999) (Supplementary Table S1).

Several nonsense variants are predicted to lead to an early termination after amino acids 5, 9, 20, 47, 61 or 101. Like with OCNDS, there is heterogeneity in the clinical phenotypes. No *CSNK2B* full gene deletion mutant has yet been described. Missense and other in-frame variants are found along the whole protein sequence: one patient carries an in-frame duplication p.(G27D28dup) in the highly acidic N-terminal domain of CK2β (AA 1–104) which contains docking sites for various proteins (Bojanowski et al., 1993; Appel et al., 1995; Li et al., 1997; Romero-Oliva and Allende, 2001; Theis-Febvre et al., 2003; Tapia et al., 2004). The patient has mild ID and GTCS seizures (Table 2B and Supplementary Table S2). Seven patients were described having variants in the KEN box, a sequence motif with the consensus KENxxxN (AA 32–40) that is targeted by the ubiquitin protein ligase APC (Pfleger and Kirschner, 2000). All of them presented with delayed speech, dysmorphic features and mild/moderate ID, four with seizures, but none of them with GTCS (Table 2B and Supplementary Table S2). Another motif, the D- or destruction box-like motif analogous to sequences found in cyclins (AA 47–55), that is recognized by the ubiquitin proteolysis machinery (Allende and Allende, 1995), was the location of variants in two POBINDS cases (Table 2B and Supplementary Table S2). However, the variants are predicted to lead to premature termination of the polypeptide chain, and therefore the phenotype of the patients cannot inform us about the functional effect of a D-box variant. The acidic loop (AA 55–64) can bind to the basic cluster of the CK2α subunit that it important for substrate binding and recognition. Thus, the acidic loop could be seen as pseudo-substrate region, that competitively hinders the access of negatively charged substrates to the active site, thereby essentially down-regulating CK2 activity (Boldyreff et al., 1994; Marin et al., 1997). The acidic loop is also necessary for the creation of inactive supramolecular structures of CK2 (Lolli et al., 2017). One *de novo* nonsense variant p.(E61*) has been described in the acidic loop resulting in the insertion of a premature stop codon at p.61. Four missense variants were described further C-terminal, in a region that has been shown to interact with Topoisomerase II (AA 51–110) (Leroy et al., 1999), with clinical phenotypes varying from mild to severe, from absence to presence of GTCS.

**TABLE 2B:** POBINDS patients and their symptom profiles.

	Structural domain	**N-Term**					**Zinc finger domain**												
																					Cys 109	**Cys 114**		**Cys 137**			**Cys 140**				**Positive regulatory region**	**C-Term**	
								**KEN Box**					**D box**	**Acidic loop**	**Topoisomerase II interaction site**							**p53 interaction site**											
	Variant p.(...)	M1?	G5*/ R86C	W9*	E20*	25Mfs*1	27D28du	D32N	F34S	N35K	T37Yfs	Q42*	R47*	E61*	E77K	R86C	I88Ifs*46	M97I	L98Afs*1	Y101*	pF106V	R111P	M132fs	C137R	C137G	C137F	H165R	167Sfs*6	179Yfs*4	N181fs	L187R	207Ffs*3	Splic site variants
	Growth delay	+	+	?	?	?	?	+/?	?	+	-	?	+/?	+	+	+/?	+	?	?	+	+	+	+	?	+	+	+	+	+	+	+	+	+/-
Developmental delay	Motor: walking onset (mo)	23	+	+	17	18	15	+	15	+	17	+	+	+	21	+	-	17	24	+	17	+	17	?	-	+	+/?	-	?	+	+	16.5	+
	Speech onset (mo)	28	?	+	+	?	21	+	+	+	18	?	+	+	21	+/?	-	36	?	+	+	+	36	24	-	+	+/?	+	?	+	?	+	+
Dysmorphic features		+/-	?	?	?	?	?	+	+	+	-	?	+/?	+	+	?	?	+	+	+	+	?	+	+	?	?	+/?	?	-	+	?	?	?
ID/LD		+/?	+	+	+	?	+	+	+	+	?	+	+	+	+	+/-	-	+	+	+	+	+	+ Mild	?	-	+	+/?	+	+	+	+	+	+/?
Neurological and behavioural problems	Hypotonia	+/?	?	+/?	?	?	-	+/?	?	-	?	?	+/?	+	+	-/?	?	?	+	+	+	?	Severe	?	?	?	+/?	?	?	+	?	?	+/-/?
	Autistic features	+/?	?	+/?	+	?	-	+/-	+	-	?	?	+/?	+	?	-/?	?	?	+	?	?	?		+	?	?	?	?	?	+	?	?	-/?
	Stereotyped behaviour	?	?	?	?	?	-	-/?	?	-	?	?	?	?	?	-/?	?	?	?	?	?	?		?	?	?	?	?	?	?	?	?	+/-/?
	Hyperactivity	?	?	?	?	?	-	-/?	?	-	?	?	?	?	?	-/?	?	?	?	?	?	?		?	?	+/?	?	?	?	?	?	?	+/-/?
	ADHD features	+/?	?	?	?	?	-	-/?	?	-	?	?	?	?	?	-/?	?	?	+	?	?	?		?	?	+/?	?	+	?	?	?	?	+/-/?
	Seizures	+/-	+ GTCS	+	+ GTCS	+	+ GTCS	+	-	-	+	+	+	+ GTCS	-	+	+ GTCS	+ GTCS	?	+	+	+ GTCS	+ GTCS	+ GTCS	+ GTCS	+	+	+	+	+ GTCS	+	+ GTCS	+
Problems	Sleep	?	?	?	?	?	?	?	+	?	?	?	?	?	?	?	?	?	?	?	?	?	?	?	?	?	?	?	?	?	?	?	?
	Eating/gastro intestinal	?	?	+/?	+	?	?	?	?	?	?	?	+/?	+	?	?	?	?	?	?	?	?	?	?	?	?	+/?	?	?	?	?	?	?
Anomalies	EEG	+/?	-	+/?	?	+	?	?	?	?	+	?	+/?	?	?	?	-	?	-	?	?	-	?	?	+	+/-	+/-/?	-	?	?	+	+	+/-/?
	MRI	+/?	+	-/?	?	-	+	+/?	+	?	-	?	-/?	?	?	?	-	?	-	?	?	-	?	?	-	-	+/-	-	+	?	-	+	+/-/?

All patients whose symptoms were published thus far are ordered by the position of their amino acid alteration, from left to right: N-terminus to the C-terminus. Patients with the same variant are grouped in one column. “+” indicates that symptom is present, “−” that it is absent, “+/−” is used when patients were described who had or had not the specific phenotype, “?” is used when the specific phenotype was not discussed in the corresponding publication. When possible, age indications (in months) for symptom onset/detection are given. Abbreviations: ATP: adenosine triphosphate; GTP: guanosine triphosphate; ID: intellectual disability; LD: learning disability; ADHD: attention deficit hyperactivity disorder; GTCS: generalized tonic-clonic seizure; GERD: gastroesophageal reflux disease; G-tube: Gastrostomy tube; EEG: electroencephalography; MRI: Magnetic resonance imaging. For more details, Supplementary Table S2. Information derived from (Sakaguchi et al., 2017; Li et al., 2019; Nakashima et al., 2019; Bonanni et al., 2021; Ernst et al., 2021; Wilke et al., 2022).

The central part of CK2β protein consists of the juxta-dimer interface region, which contains the zinc-finger region (AA 105–146). Four conserved cysteine residues (C109, C114, C137 and C140) coordinate Zn^2+^ ions, which are necessary for β-β subunit dimerization (Chantalat, 1999). Nine patients exhibit variants in this region. Variant at position C137 is present in four patients and thus could be considered a variant hotspot (Table 2B and Supplementary Table S2). The clinical phenotype is relatively homogeneous: 8/9 patients were reported with delayed growth, and 7/9 with various degrees of motor and speech development delay, 4/9 with mild dysmorphic features and 6/9 with mild ID. All nine patients have seizures, with 7/9 presenting GTCS. Two patients with the missense variant p.(R111P) (Li et al., 2019) were diagnosed with motor and speech delay, ID and GTCS. The C-terminal region (starting at AA 171), containing the positive regulatory region, contributes to CK2β dimerization but is also necessary for interaction with CK2α/α’ (Meggio et al., 1995; Marin et al., 1997; Chantalat 1999; Sarno et al., 1999; Niefind et al., 2001). The crystal structure of the holoenzyme indicates that the C-terminus of CK2β is in direct contact with the CK2α, stabilizes the β/β and α/β contacts and points away from the enzyme body and also stimulates kinase activity. However, in the β dimer (CK2α absent) it acts in a destabilizing manner (Niefind et al., 2001). One can therefore hypothesize that variants in the C-terminal region affect formation of a stable holoenzyme, somewhat like a variant in the zinc finger domain would do. Interestingly, all seven patients with variants in this region had ID and suffered from epilepsy, with 5/7 having GTCS. One may speculate that a deleterious effect on β/β dimerization will negatively impact on holoenzyme formation and that reduced amount of holoenzyme has a striking effect on the appearance of GTCS.

Ten patients carry seven nonsense variants [p.(G5*), p.(W9*), p.(E20*), p.(Q42*), p.(R47*), p.(E61*) and p.(Y101*)] predicted to ablate the formation of full length CK2β protein. The patients present delayed growth, motor development and speech, with two patients being nonverbal. 8/10 present ID, with three of these eight having profound ID, and 4/10 present dysmorphic features. Half of the patients present hypotonia and autistic features, and all of them suffer from epilepsy, with 3/10 having GTCS and other three presenting myotonic-atonic seizures. Interestingly, four patients present eating/gastrointestinal issues, symptoms that are more often found in OCNDS patients.

## Effect of Variants on CK2 Activity

The most obvious result of any pathogenic variant in all CK2 subunits that does not abolish protein expression/translation, is to alter the activity of an enzyme, either by enhancing or, more probably, reducing it. Our group has studied the *in vitro* activity of 16 different *CSNK2A1* missense mutants and found that the activity towards a consensus peptide is significantly reduced for all. This is the case when CK2α proteins are bacterially expressed, purified, and tested in the presence and absence of purified CK2β, or when mutants are overexpressed in mammalian cells and immunoprecipitated (Dominguez et al., 2021). However, it has been recently suggested that the *CSNK2A1* p.(K198R) variant does not lead to a generic reduction in overall activity, but to a change in substrate specificity towards reduced preference for acidic residues at position +1, for T as phosphoacceptor and a novel preference for Y (Caefer et al., 2021). This hypothesis, generated on the basis of mass spectrometry of bacterial lysates expressing the CK2 mutant, however, awaits confirmation in mammalian cells since the bacterial phosphoproteome may not correctly reflect the situation in mammalian cells, where several levels of CK2 activity regulation exist, starting from holoenzyme and multi-protein-complex formation, substrate recruitment, compartmentalization within a cell, and, in particular, post-translational modifications, such as hierarchical phosphorylation.

It is very surprising to observe no loss-of-function variant such as nonsense, frameshift and only two splice site consensus variants. However, taking into consideration the one OCNDS patient with full gene loss and one patient with pathogenic variant of the active site p.(D156H), it is plausible that a loss-of-function rather than a gain-of-function mechanism is present.

Whether missense variants could act in a dominant-negative manner, by competitively binding to *in vivo* substrates, or by preventing the formation of an active holoenzyme, or whether a haplo-insufficient effect underlies the phenotypes whereby the reduced amount of active wildtype CK2α is insufficient, cannot be clearly stated at this point. It is also possible that both mechanisms are at play, and even that different mutants exert their effects through either mechanism.

Could there be compensatory upregulation of wild type CK2α, CK2α’ or CK2β in OCNDS and POBINDS? We have tested the expression of both catalytic isoforms in OCNDS patient-derived fibroblasts [CK2α p.(R47G), p.(D156E) and p.(K198R)] and have not detected enhanced amounts of CK2α or α’ in patient fibroblasts compared to parental control lines. In contrast, we found that CK2β protein is upregulated in these lines (Dominguez et al., 2021). Such regulation however could be cell-type dependent and thus further studies should shed light on this question.

### CK2β-dependent Mechanisms

CK2β modulates CK2’s biological functions through enabling the formation of a holotetramer (Boldyreff et al., 1996) (1), formation of higher-order multimers (Lolli et al., 2012) (2), recruitment of substrates (Guerra and Issinger, 1999) (3) and modulation of activity towards certain substrates (Meggio et al., 1992) (4). Finally, it is important to note that CK2β also has roles in the cell that are independent of CK2α (Guerra and Issinger, 1999) (5).

1) The zinc finger domain mediates β-β dimerization which can occur in the absence of CK2α and is a prerequisite for the incorporation of catalytic CK2 subunits into tetrameric complexes (Niefind and Issinger 2005). The CK2β homodimer is the building block for the holoenzyme, by bridging the two catalytic subunits. Thus, CK2β missense variants within the zinc finger motif, especially the conserved cysteines, may result in loss of CK2β dimers and ultimately, CK2 heterotetramers. The importance of CK2β dimerization was demonstrated in *Drosophila* by expression of mutagenized CK2β transgenes in a CK2β null mutant background. Variants of either cysteine residue pair (109/114 or 137/140) resulted in a CK2β protein which was unable to rescue the lethality of the CK2β null mutant (Canton et al., 2001). Generally, in POBINDS patients, variants in the zinc finger domain and/or the C-terminal domain seem to have a severe phenotype. Some variants in the more N-terminal region do not show ID and/or epilepsy. For example, 100% of the patients with missense variants in the acidic loop or more N-terminal to it, do not exhibit GTCS (Supplementary Table S2). However, variants in the more central region, within the Topoisomerase interaction region, or more or C-terminal to it are generally characterized by seizures, mainly of GTCS type (Supplementary Table S2).
2) Supramolecular assemblies of CK2 that are driven by inter-molecular interactions between the acidic loop of CK2β and the p+1 loop of CK2α of two different holoenzymes (Lolli et al., 2012) were identified. They are thought to be inactive forms that, when needed, can disassemble into the active tetrameric form of CK2. These oligomers form *in vitro* at low salt concentrations in linear or circular organization complexes (Niefind and Issinger, 2005), but also in *E. coli* as shown by native mass spectrometry (Seetoh et al., 2016). 12 patients with variants in the CK2α p+1 loop are described in the literature [9 of which have the p.(K198R) variant], and it would be interesting to determine in patient-derived cells whether the formation of CK2 multimers is present and altered.
3) CK2β confers to the holoenzyme the ability to interact with certain substrates, such as p53 (Appel et al., 1995) and topoisomerase II (Bojanowski et al., 1993). The purified isolated CK2α subunit by itself is unable to bind to both enzymes, thus the CK2β subunit mediates the recruitment, which, for interaction with p53 has been narrowed down to CK2β AA 72-149 and for topoisomerase II to AA 51-110, a region in which several POBINDS-linked variants are present. Phosphorylation of p53 at position serine 392 by CK2 activates the site-specific DNA-binding function and tetramerization of p53. Mice expressing p53 proteins p.(S389A), the mouse equivalent of human S392 exhibit susceptibility to various tumors (Meek and Cox, 2011). A KSSR motif (AA 147–150), located at the interface of the β-β dimer, has been shown to be required for two other CK2 substrates, the Epstein-Barr virus EBNA1 protein and C18orf25/ARKL1 (Cao et al., 2014), however no such variants have been identified in POBINDS patients.

Another relevant example of CK2β dependent substrate recruitment is the transcription factor Olig2 in the developing mouse brain (Xu et al., 2020). Disruption of CK2β leads to inhibition of neuronal stem cell proliferation and loss of differentiation in oligodendrocyte precursor cells (OPCs) in mice. Olig2 is required for OPCs development and was identified *in vitro* as a strict CK2β-dependent CK2 substrate (Huillard et al., 2010). Other binding partners of CK2 that are recruited *via* the β isoform are discussed in the review by Bibby and Litchfield (Bibby and Litchfield, 2005).

4) CK2β modulates kinase specificity towards CK2 substrates. *In vitro* studies, performed in the presence or absence of CK2β, generally showed that some substrates are phosphorylated equally with or without CK2β while others are less phosphorylated in the absence of CK2β (Meggio et al., 1992). Only a limited number of protein substrates are phosphorylated by CK2α alone but not the holoenzyme, such as Calmodulin (Meggio et al., 1992). Presumably, in these instances, the β subunit does not turn off catalytic activity, since CK2 is constitutively “on”, but mediates its effect through specific interactions with the protein substrates (Pinna, 2002). Phosphorylation of Calmodulin by CK2 alters the interaction with various downstream effectors such as CaM-dependent cyclic nucleotide phosphodiesterase, Ca^2+^-ATPase, Ca^2+^/CaM-dependent protein kinase II, myosin light chain kinase, and NO synthase (Arrigoni et al., 2004). It could be hypothesized that POBINDS mutants that are expected to prevent holoenzyme formation, will elevate the proportion of CK2α monomers over the holoenzyme and alter CaM phosphorylation and its function. This may have an impact on several neural processes, such as synaptic plasticity via glutamate receptor homeostasis (Sanz-Clemente et al., 2013).
5) Recently, muscle cell clones (C2C12) that are devoid of either α, α’ or β subunits were generated, and phosphoproteomic analysis revealed that lack of the β subunit affected grossly the same phosphosites than knockout of CK2α/α′, however many phosphosites that do not conform to the CK2 consensus were also altered, strengthening the argumentation for roles of CK2β that are independent of CK2 activity (Borgo et al., 2019). CK2β dimers, in the absence of CK2α, were found in mouse testis and brain (Guerra and Issinger, 1999) and appear to have regulatory function on several other protein kinases, such as A-Raf (Hagemann et al., 1997), c-Mos (Chen et al., 1997) and Chk1 (Guerra et al., 2003). A-Raf belongs to a family of cytoplasmic S/T protein kinases within the MAPK pathway (Chong et al., 2003). In two independent yeast screens (Boldyreff et al., 1996; Hagemann et al., 1997), CK2β was found to specifically interact with A-Raf, which resulted in a 10-fold enhancement of its activity towards MEK in co-expression studies in insect cells. Interestingly, expression of CK2α results in abolished MEK activation observed in the presence of CK2β, suggesting that CK2α might be competing with A-Raf for binding to CK2β (Hagemann et al., 1997). CK2β, by direct binding, inhibits Mos, an activator of MAPK, that is highly expressed in germ cells (Sagata et al., 1988). CK2β binds Mos via its C-terminus, leading to reduced MAPK activation (Chen et al., 1997; Lieberman and Ruderman, 2004). It was suggested that regulation of Mos activity by CK2β occurs during early stages of *Xenopus* oocyte maturation, and that, during later developmental stages this inhibition abates due to upregulated expression of Mos molecules that outnumber CK2β molecules (Chen et al., 1997). ChK1 is a cell cycle checkpoint kinase (G2) that is required for ES cell viability (Takai et al., 2000). CK2β binds ChK1 via its C-terminus *in vitro* and *in vivo*, to activate ChK1 activity (Guerra et al., 2003).

## CK2-dependent Biological Processes That May Underlie Symptoms

Based on our mass spectrometry results using patient fibroblasts (Dominguez et al., 2021), we hypothesize that OCNDS-linked variants will lead to overall reduced phosphorylation of *in vivo* substrates, of which several have been linked to functions such as synaptic transmission and plasticity, neuritogenesis which are crucial for neural development and homeostasis, as reviewed (Blanquet, 2000; Castello et al., 2018). In this section, we will discuss pathways that involve CK2 activity and appear most pertinent in respect to patient symptoms.

1) Changes in cell growth and apoptosis pathways affect growth and morphogenesis

CK2 is implicated in the Akt/GSK3β pathway, an anti-apoptotic, pro-survival pathway that is important in tumorigenesis and tumor growth by directly phosphorylating Akt at position S129 in immortalized mammalian cells (Di Maira et al., 2005). Recently CK2, especially the CK2β and α’ subunit, have been attributed a role in cell migration and adhesion (Lettieri et al., 2019). In the brain, by interaction with mammalian/mechanistic target of rapamycin (mTOR), Akt regulates neuronal processes like morphogenesis, synapse formation, plasticity, and dendritic development (Hers et al., 2011). Since autism spectrum disorders have been associated with alterations in brain connectivity in mouse models and autistic children (Ellegood et al., 2015), it could be possible that CK2α variants cause modifications in the Akt pathway that could contribute to the ASD-like symptoms in OCNDS. In OCNDS-derived fibroblasts, however, we did not detect a reduction of pS129 Akt or pS473 Akt which suggests, again, that the regulation of signaling pathways may be cell-type dependent and different in immortalized versus primary cells (Dominguez et al., 2021).

As cells rapidly undergo mitosis during neural development, it is as important that a controlled portion of cells undergoes apoptosis, in a process called pruning, and the balance between these processes underlies correct neural and organ development (Putcha and Johnson, 2004). A role of CK2 in the cell cycle was first deduced due to cell-cycle dependent phosphorylation of CK2α and CK2β (Litchfield, 2003). To date, many more substrates and binding partners, such as p53, Akt, topo2, clearly involve CK2 in both of these processes (Filhol et al., 1992; Bojanowski et al., 1993; Di Maira et al., 2005).

Since a detailed discussion of CK2’s role in apoptosis and the cell cycle would be too lengthy here, we refer to reviews on cell cycle regulation (St-Denis et al., 2009), survival and apoptosis (Duncan et al., 2010; Hanif and Pervaiz, 2011).

Wnt signaling is an important regulator of development, acting through a canonical and a non-canonical pathway to affect cell fate determination, polarity, and early morphogenetic movements. CK2 has been shown to modulate Wnt signaling in *Drosophila* and mammalian cells, since CK2 phosphorylates and stabilizes Dbl (downstream of Wnt-activated frizzled receptors), the transcriptional co-factor β-catenin and the transcription factor TCF/LEF itself, leading to the transcriptional activation of target genes (Song et al., 2003; Seldin et al., 2005). Interestingly, functional coupling of Wnt3a to Frizzled-1 receptor produces transient enhanced activity of CK2 and increased accumulation of β-catenin (Gao and Wang, 2006). Thus, a reduction in CK2 activity may lead to reduced target gene expression and improper development. Canonical Wnt signaling in the ventral diencephalon regulates the formation of the pituitary gland (Osmundsen et al., 2017), which could explain the abnormalities found in four MRI out of the 35 OCNDS patients described (MRI was taken in 23 patients). If the pituitary gland is affected in OCNDS, it could lead to altered secretion of hormones, such as the growth hormone (GH) (Chinoy and Murray, 2016), resulting in retarded growth.

Recently, CK2α has been linked to trafficking of cilia, microtubule-projections mediating morphogenic and mitogenic signals during development, that, when dysfunctional, cause ciliopathies characterized by intellectual disability and brain malformations (Valente et al., 2014). CK2α localizes at the mother centriole and mediates cilia structure and stability. It interacts with a key regulator of ciliogenesis, the kinase TTBK2. Expression of OCNDS-linked mutants *CSNK2A1* p.(R80H), p.(D156H) and p.(R191Q) mutants results in structural defects of cilia in mouse embryonic fibroblasts (MEFs) (Loukil et al., 2021). It still remains to be determined if this effect is dependent on CK2 activity, and, if yes, which substrates mediate this effect.

2) Changes in synaptic plasticity affect motor abilities, learning/memory and seizure propensity

CK2 is not only localized to the nucleus and cytoplasm of neurons, but was also detected at the plasma membrane (Rebholz et al., 2009), more precisely at the post-synaptic density in rat hippocampal and cortical preparations (Soto et al., 2004). CK2 activity is enriched in synaptosomes (Girault et al., 1990) and a whole set of CK2 substrates identified *in vitro* or *in vivo* clearly link CK2 to the control of synaptic activity, as discussed in a previous review (Castello et al., 2017). CK2α modulates the homeostasis of neurotransmitter receptors, such as ion channel receptors (Montenarh and Götz, 2020) and GPCRs that are coupled *via* Gα_s_ (Castello et al., 2018). As an example of an ion channel, the NMDA glutamate receptor, a cation channel for Ca^2+^, Na^+^ and K^+^ with crucial roles in synaptic plasticity, memory, and learning, shall be mentioned here. CK2 phosphorylates the NR2B subunit of the NMDAR, leading to a disruption of the interaction with PSD-95 and to decreased receptor surface expression in neurons (Chung et al., 2004), in a process driven by synaptic activity and CamKII (Sanz-Clemente et al., 2013). This seems to be of a particular importance during mouse development, in the early postnatal period, where CK2-mediated NR2B-endocytosis resulted in a switch from NR2B to NR2A expression at cortical synapses (Sanz-Clemente et al., 2010). Thus, the integrity of such synapses might be compromised due to insufficient CK2 activity.

Seizures are disorders of neuronal network excitability, which is accompanied by pronounced changes in intracellular and extracellular ion concentrations involving a multitude of ion channels (Raimondo et al., 2015). SK channels provide the hyperpolarizing K^+^ conductance that is fundamental for a wide range of physiological processes, including neuronal excitability (Stocker et al., 1999). They are gated by Ca^2+^ ions *via* the Ca^2+^ censing protein calmodulin that is bound to the intracellular C-terminal chain of the SK channel. CK2 has been detected in complex with calmodulin, to phosphorylate it at T80 and reduce its Ca^2+^ sensitivity, thereby accelerating SK channel deactivation (Bildl et al., 2004). Indeed, a CK2 inhibitor (TBB) enhanced K^+^ currents and hyperpolarization in a seizure model (Pilocarpine) and blocked spontaneous epileptic activity in an acute slice model (Brehme et al., 2014). This finding, on the first glance seems at odds with the high seizure incidence in POBINDS patients, however, TBB acts on CK2 kinase activity, whereas we hypothesize that it is plausible for the high seizure incidence in POBINDS to be caused by an activity-independent mechanism. Several other ion channels (Ca^2+^, Na^+^, Cl^−^, K^+^) have been shown to be CK2 substrates or binding partners and altered function of these channels will affect physiological neuronal excitability, and may result in neurological disorders such as epilepsy (Montenarh and Götz, 2020).

Another link between CK2 and neural network synchrony and epilepsy is the Mdm2-p53-Nedd4-2 pathway. Both p53 and Mdm2 are CK2 substrates (Filhol et al., 1992; Allende-Vega et al., 2005), and inhibition of CK2 leads to enhanced p53 activity (Dixit et al., 2012). In a kainic acid-induced seizure model in mice, inhibition of p53 reduced seizure susceptibility through modulation of neural network synchrony (Jewett et al., 2018). In the context of OCNDS/POBINDS, it is plausible to speculate that reduced CK2 activity therefore could exert an enhancing effect in the kainic acid seizure model.

In the case of GPCRs, reduced CK2 activity is predicted to delay agonist-induced desensitization and endocytosis of these receptors, similarly to what was observed for dopamine D1 and serotonin HTR4 receptors after knockdown or pharmacological inhibition of CK2 (Rebholz et al., 2013; Castello et al., 2018). A role for the dopamine D1 receptor in the OCNDS/POBINDS phenotype is further made conceivable by the motor behaviors of conditional Drd1a-Cre CK2 KO mice: they are hyperactive and exhibit stereotypies, and both phenotypes are normalized upon administration of D1 antagonist SCH23390. Furthermore, these mice also have defects in motor performance and learning in the rotarod.

3) Hypotonia

A majority of patients (OCNDS: 77%; POBINDS 75%, Tables 2A, 2B) suffers from hypotonia early in life, which contributes to feeding difficulties as well as delays in motor development. CK2 phosphorylates or interacts with several proteins that are involved in myogenesis or play a role at the neuromuscular junction, as recently reviewed (Hashemolhosseini, 2020).

CRISPR-mediated knockdown of either the catalytic or the regulatory subunits showed that in particular the lack/absence of CK2β severely impairs the growth of C2C12 cells (Borgo et al., 2017, 2019), hinting towards an important role for CK2β in muscle cells. Interestingly, CK2β conditional knockout mice with CK2β lacking in skeletal muscle display reduced muscle strength. Skeletal muscle cell lysates derived from these mice have reduced activity towards Tomm22, a component of the translocase complex of the outer mitochondrial membrane, termed Tomm complex, paralleled by enhanced mitochondrial degradation through mitophagy. Phosphorylation of Tomm22 in a CK2β-dependent manner thus protects mitochondria in skeletal muscle from degradation (Kravic et al., 2018).

Myosins are a family of actin-binding cytoskeletal motor proteins that, as a complex of heavy and light myosin chains, hydrolyze ATP during muscle contraction. During myogenesis, myosins need to assemble into long thick filaments. It was shown that phosphorylation of myosin-IIA heavy chain by PKC or CK2 inhibits the assembly of into filaments. CK2 phosphorylation of the myosin-IIA heavy chain reduced binding of the Mts1 calcium-binding protein, thereby inhibiting mts1-induced filament disassembly and assembly (Dulyaninova et al., 2005).

CK2-dependent phosphorylation is important for myogenesis and muscle homeostasis. CK2 is present at the neuromuscular junction to regulate acetylcholine receptor stability, as recently reviewed (Hashemolhosseini, 2020). Conditional CK2β KO mice with lack of CK2β in skeletal muscle showed reduced muscle strength and abnormal metabolic activity of oxidative muscle fibers. This was linked to deficient phosphorylation of an outer mitochondrial membrane protein, Tomm 22 (Kravic et al., 2018). CK2α was further found to be involved in activation of muscle-specific genes, as its inhibition leads to a significant reduction in muscle-specific genes in C2C12 cells (Salizzato et al., 2019).

Taken together, CK2 activity is necessary for muscle genesis and homeostasis, both of which could be impacted by variants of either of the CK2 subunits.

4) Autistic features

55% of OCNDS and 56% of POBINDS patients have been diagnosed with ASD, and several fields of study, from genetics to biochemistry, deliver arguments for a role of CK2 in this disorder. ASD has both genetic and environmental origins. One predisposing environmental factor is the prenatal exposure to valproic acid (VPA) that increases the risk of ASD in children (Nicolini and Fahnestock, 2018). In rats, CK2α was found to be upregulated after prenatal VPA exposure, however these results are based solely on western blotting and require further confirmation (Santos-Terra et al., 2021).

We already mentioned that in Drd1a-Cre conditional CK2 KO mice dopamine D1 receptor signaling is upregulated and that endocytosis of this receptor is modulated by CK2 (Rebholz et al., 2013). Indeed, several genes of the DA network have been linked to ASD, such as the genes encoding syntaxin 1 (STX1) (Nakamura et al., 2008) or dopamine transporter (DAT) (Hamilton, 2013). Autism-associated variants of these two genes show decreased phosphorylation of STX1 (at S14) by CK2, resulting in reduced STX1/DAT interaction and disruption of the reverse transport of DA (Cartier-Z et al., 2015). This functional interaction was tested in the locomotive response to amphetamine in *Drosophila*. Both, STX1A-R26Q and hDAT-R51W variants responded less to amphetamine, similarly to *Drosophila* expressing a dominant negative form of CK2 (Cartier-Z et al., 2015).

It is known that CK2 activity alters transcription via phosphorylation of a set of transcription factors such as e.g. TFIIA, IIE, or IIF, as reviewed in (St-Denis et al., 2009). The protein encoded by the ASD susceptibility gene AUTS2 was found bound to CK2β within the large Polycomb Repressive Complex 1 (PRC1) (Gao et al., 2014). This complex normally catalyzes the monoubiquitination of histone H2A (at K119) and leads to compaction of chromatin and transcriptional repression. CK2, through phosphorylation of another member of this complex, RING1B, inhibits PRC1-AUTS2-mediated monoubiquitination of H2A, thereby turning a transcriptional repressor into an activator and affecting the transcriptional profile of cells (Gao et al., 2014).

On the level of translation, the fragile X mental retardation protein (FMRP) is a mRNA-binding translational repressor that associates with 4–6% of brain transcripts, with autism risk gene transcripts being overrepresented. Absence or severe reduction of FMRP are responsible for fragile X syndrome, the most common monogenic cause of autism spectrum disorder (Verkerk et al., 1991). CK2 phosphorylates murine FMRP at the site S499, a site which is required for its repressor activity, and thereby primes for further phosphorylation at nearby sites by other kinases (Bartley et al., 2016).

## Conclusion

OCNDS and POBINDS are two distinct newly described NDDs with causative variants in the genes coding for Csnk2a1 and Csnk2b. The symptom overlap is large, and the most striking difference is the elevated propensity to seizures in POBINDS. CK2β has specific roles in the cells, such as regulating kinases other than CK2, that could be at the origin of the seizure phenotype in POBINDS.

Due to its ubiquitous expression (https://www.proteinatlas.org/ENSG00000101266-CSNK2A1/tissue), its promiscuity, based on a non-stringent consensus (S/TxxD/E) (Meggio et al., 1994), it is most plausible that CK2 acts through many pathways and substrates to lead to the symptom profiles of both NDDs.

Clearly, experimental studies are missing and through the use of patient derived cells, especially iPS cells, as well as mouse models of both diseases more mechanistic knowledge has to be obtained. These models can also be used for the search of potentially druggable targets and to test therapeutic approaches that could be transferred from treatment of other NDDs.

## Supplementary Material

Supplementary Table S1 Detailed table of OCNDS patients and their symptom profiles. All patients whose symptoms were published thus far are ordered by the position of the gene variation, position within the gene structure as well as the effect on amino acid sequence, as far as it could be predicted. More detailed information about the different symptoms is given in this table. Abbreviations: ATP: Adenosine triphosphate; GTP: Guanosine triphosphate; yo: years old; mo: months old; ID: Intellectual Disability; LD: Learning disability; ADHD: Attention deficit hyperactivity disorder; GTCS: generalized tonic-clonic seizure; GERD: Gastroesophageal reflux disease; G-tube: Gastrostomy tube; EEG: Electroencephalography; MRI: Magnetic resonance imaging.

Supplementary Table S2 Detailed table of POBINDS patients and their symptom profiles. All patients whose symptoms were published thus far are ordered by the position of the gene variation, position within the gene structure as well as the effect on amino acid sequence, as far as it could be predicted. More detailed information about the different symptoms is given in this table. Abbreviations: D Box: Destruction box; yo: years old; mo: months old; ID: Intellectual Disability; LD: Learning disability; ADHD: Attention deficit hyperactivity disorder; GTCS: generalized tonic-clonic seizure; GERD: Gastroesophageal reflux disease; G-tube: Gastrostomy tube; EEG: Electroencephalography; MRI: Magnetic resonance imaging.
