# Supplementary material for: Comparing Two Neurodevelopmental Disorders Linked to CK2: Okur-Chung Neurodevelopmental Syndrome and Poirier-Bienvenu Neurodevelopmental Syndrome—Two Sides of the Same Coin?
Source: Front Mol Biosci. 2022 May 26;9:850559. doi: 10.3389/fmolb.2022.850559 (PMC9182197; doi:10.3389/fmolb.2022.850559)
Supplement: Supplementary file 1 [file Table1.pdf]

Protein structure	N-term		ATP/GTP Binding domain (AA 45-53)								Basic cluster (AA 68-8	
	Patient E3	Patient E2	Patient A1	Patient C1	Patient E6	Patient A5	Patient H	Patient M	Patient E8	Patient C2	Patient E5	Patient N2
Publication	Chiu et al., Patient 3	Chiu et al., Patient 2	Okur et al., Patient 1	Owen et al., Patient 1	Chiu et al., Patient 6	Okur et al., Patient 5	Martinez-Monseney et al., Patient 1	Wu et al., 2020 Patient 1	Chiu et al., Patient 8	Owen et al., Patient 2	Chiu et al., Patient 5	Wu et al., 2021 Patient 2
Amino acid change	p.(M1?)	p.(E27K)	p.(R47Q)	p.(R47Q)	p.(R47Q)	p.(Y50S)	p.(Y50C)	p.(Y50C)	p.(S51R)	p.(S51N)	p.(V73E)	p.(R80C)
Variant	c.1A>G	c.79G>A	c.140G>A	c.140G>A	c.140G>A	c.149A>C	c.149A>C	c.149A>C	c.151A>C	c.152G>A	c.218T>A	c.238C>T
Heritability	*De novo*	*De novo*	*De novo*	*De novo*	*De novo*	*De novo*	*De novo*	*De novo*	*De novo*	*De novo*	*De novo*	*De novo*
Classification	Start loss	Missense	Missense	Missense	Missense	Missense	Missense	Missense	Missense	Missense	Missense	Missense
Consequence prediction	?	?	?	?	?	?	?	?	?	?	?	?
Position	Exon 3	Exon 3	Exon 4	Exon 4	Exon 4	Exon 4	Exon 4	Exon 4	Exon 4	Exon 4	Exon 5	Exon 5
Sex	Male	Male	Female	Male	Female	Female	Female	Male	Male	Female	Male	Female
Age at last follow-up	14 yo	4 yo	6 yo	6.1 yo	2 yo	2 yo	5.8 yo	8 yo	4 yo	11.1 yo	8 yo	2 yo
Growth problems	None	Microcephaly, short stature	Microcephaly, short stature	Short stature, underweight	Microcephaly, short stature	Short stature, underweight	Short stature, delayed bone age	Delayed growth	Short stature, underweight	Short stature, underweight	Microcephaly, short stature	Short stature, underweight, microcephaly
Motor development	N/A	Walked at 22 mo	Sat at 15 mo, Walked at 28 mo	Sat at 6 mo, Walked at 22 mo	Walked at 6 yo	Sat at 8 mo, Walked at 20 mo	Sat at 10 mo, Walked at 28 mo	Delayed	Walked at 30 mo	Sat at 7 mo, Walked at 24 mo	Delayed	Cannot sit or stand without support
Speech development	N/A	First words at 3 yo	200 words	Lost at 24 mo	No recognizable speech	2 words at 1 yo	Poor speech	Delayed	Only single words	First words at 36 mo	Delayed	No meaningful words
Dysmorphic features	N/A	Straight horizontal eyebrows and broad nasal bridge	Hypertelorism, low set folded ears, high palate, high arched eyebrows	N/A	Frontal bossing, conjunctival grey spots, small mouth	Extra folds in helix, inverted epicanthal folds, broad great toes	Midline defects as flat face, arched eyebrows, down-slanting palpebral fissures with epicanthus, wide philtrum, bifid tongue	N/A	Prominent forehead, arched eyebrows, low set ears, retrognathia	N/A	N/A	Arched eyebrows, epicanthic folds, broad nasal bridge, micrognanthia
ID	Yes	N/A	Yes	N/A	Yes	Yes	N/A	Yes	N/A	N/A	Severe	N/A
Neurological and Behavioural problems	Hypotonia, ASD, dyspraxia	Autistic traits, stereotypic movements, tactile hypersensitivity	Hypotonia, Ataxia, tantrums, ADHD features	None	Hypotonia	Hypotonia	Wave-hands stereotypes with excitation and diurnal bruxism	breath-holding spells	Hypotonia, hyperkinesia, temper tantrums, low attention span, bruxism	None	N/A	N/A
Seizures	N/A	N/A	No	No	N/A	Present	N/A	N/A	Myoclonic, required VPA	No	N/A	N/A
Sleeping problems	Yes	Yes	No	N/A	N/A	No	No	N/A	In infancy	N/A	Until he was 5	N/A
Eating/Gastro intestinal problems	Yes	Yes	No	Swallowing problems, GERD	Yes	Constipation, diarrhea, delayed swallowing initiation	Cannot masticate	N/A	Picky eater	No	Severe feeding difficulties, GERD	N/A
EEG	N/A	N/A	N/A	N/A	N/A	Normal	N/A	N/A	N/A	N/A	N/A	N/A
MRI	Normal after ventriculoperitoneal shunting	N/A	Pachygria	Normal	Vermis hypoplasia and thin corpus callosum	Normal	Duplication of pituatury gland	N/A	N/A	N/A	Normal	Dysplasia of corpus callosum
Others				Flexation deformity of 2nd-3rd fingers	Joint laxity	Loose joints				Severe scoliosis		

Kinase domain (AA 39-324)													
						P+1 loop							
0)	Active Site (AA156)			Activation segment (AA 175-201)									
Patient C3	Patient B	Patient N1	Patient C4	Patient A3	Patient F	Patient C5	Patient I2	Patient C6	Patient A2	Patient C7	Patient C8	Patient C9	Patient C10
Owen et al., Patient 3	Trihn et al., Patient 1	Wu et al., 2021 Patient 1	Owen et al., Patient 4	Okur et al., Patient 3	Duan et al., Patient 1	Owen et al., Patient 5	Nakashima et al., Patient 2	Owen et al., Patient 6	Okur et al., Patient 2	Owen et al., Patient 7	Owen et al., Patient 8	Owen et al., Patient 9	Owen et al., Patient 10
p.(R80H)	p.(D156H)	p.(H160R)	p.(I174M)	p.(D175G)	p.(D175G)	p.(R191Q)	p.(R191*)	p.(F197I)	p.(K198R)	p.(K198R)	p.(K198R)	p.(K198R)	p.(K198R)
c.239G>A	c.466G>C	c.479A>G	c.522A>G	c.524A>G	c.524A>G	c.572G>A	c.571A>G	c.589T>A	c.593A>G	c.593A>G	c.593A>G	c.593A>G	c.593A>G
De novo	*De novo*	*De novo*	*De novo*	*De novo*	*De novo*	*De novo*	*De novo*	*De novo*	*De novo*	*De novo*	*De novo*	*De novo*	*De novo*
Missense	Missense	Missense	Missense	Missense	Missense	Missense	Missense	Missense	Missense	Missense	Missense	Missense	Missense
?	?	?	?	?	?	?	?	?	?	?	?	?	?
Exon 5	Exon 8	Exon 8	Exon 9	Exon 9	Exon 9	Exon 9	Exon 9	Exon 9	Exon 9	Exon 9	Exon 9	Exon 9	Exon 9
Male	Male	Female	Male	Female	Male	Female	Male	Female	Female	Female	Male	Male	Female
10.7 yo	7 yo	3 yo	10.9 yo	4 yo	20 mo	10 yo	Died at 19 mo	8 yo	4.5 yo	7.3 yo	10.9 yo	18.3 yo	18.7 yo
Short stature, underweight	Microcephaly	Short stature, underweight, delayed bone age	Short stature, underweight	Short stature, underweight	Microcephaly, underweight	Short stature, underweight	Microcephaly	Short stature, underweight	Short stature, underweight	Short stature, underweight	Short stature, underweight	Short stature, underweight	Short stature, underweight
Walked at 30 mo	First steps at 22 mo	Sat at 8 mo, Walked at 18 mo	Sat at 18 mo, Walked at 30 mo	Sat at 12 mo	N/A	Sat 12 mo, Walked at 18 mo	Sat at 7 mo, Walked at 18 mo	Walked at 24 mo	Sat at 1 yo, Walked at 2 yo	Sat at 15 mo, Walked at 49 mo	Walked at 40 mo	Sat at 11 mo, Walked at 24 mo	Sat at 24 mo, Walked at 48 mo
First words at 60 mo	110 words at 3 yo	Meaningful words at 1 yo	First words at 18 mo	Not speaking yet	N/A	First words at 36 mo	One word	First words at 48 mo	Impaired, 2 words at 22 mo	First words at 48 mo	First words at 96 mo	N/A	First words at 72 mo
N/A	Microcephaly, brachycephaly	Prominent forehead, almond-shaped eyes, low-set ears	N/A	Broad nasal bridge, short upturned nose, epicanthal folds	broad nasal bridge, micrognathia	N/A	None	N/A	Round face, epicanthal folds, low set ears, high palate, arched eyebrows	N/A	N/A	N/A	N/A
N/A	Yes	Mild	N/A	Yes	N/A	N/A	N/A	N/A	Yes	N/A	N/A	N/A	N/A
Hypotonia, autistic traits, anxiety, difficult behaviors	Impaired social responsiviness, hyperactive	N/A	Hypotonia, autistic traits, mannerism, obsessions, anxiety	Stereotyped hand movements, ataxia	Hypotonia	Hypotonia, passive, easily frustrated	Hypotonia	None	Past hypotonia, volatile tantrums	Hypotonia, autistic traits, repetitive behaviors, ticks	None	Hypotonia	Hypotonia
No	No	Five febrile convulsions	No	Atonic	N/A	No	Tonic-clonic	No	No	3 Febrile	No	Absence	No
N/A	Yes	N/A	N/A	Yes	Yes	N/A	N/A	N/A	No	N/A	N/A	N/A	N/A
No	No	N/A	Swallowing problems	Constipation	Constipation, cough while eating and drinking	Swallowing problems	N/A	No	G-tube, severe GERD	No	No	No	No
N/A	N/A	Focal left temporal spike-wave discharge	N/A	N/A	N/A	N/A	N/A	N/A	N/A	N/A	N/A	N/A	N/A
Normal	Solid lesion of pineal gland	Mildly reduced pituitary gland	N/A	Simple gyral cortication	N/A	N/A	Normal	Normal	Underdeveloped left opercolum and enlarged Sylvian fissure	Normal	Normal	N/A	Delayed myelination
Hypermobile joints	Minor cystic inclusion								Gait abnormalities				

								C-term
Patient D	Patient E1	Patient I1	Patient L	Patient E4	Patient A4	Patient C11	Patient E7	Patient G
Akahira-Azuma et al., Patient 1	Chiu et al., Patient 1	Nakashima et al., Patient 1	Xu e al., Patient 1	Chiu et al., Patient 4	Okur et al., Patient 4	Owen et al., Patient 11	Chiu et al., Patient 7	Colavito et al., Patient 1
p.(K198R)	p.(K198R)	p.(K198R)	p.(K198R)	p.(P231R)	p.(?)	p.(R312W)	p.(R312Q)	p.(?)
c.593A>G	c.593A>G	c.593A>G	c.593A>G	c.692C>G	c.824+2T>C	c.934C>T	c.935G>A	c.1061-1G>C
De novo	*De novo*	*De novo*	*Vertical transmission*	*De novo*	*De novo*	*De novo*	*De novo*	*De novo*
Missense	Missense	Missense	Missense	Missense	Canonical splice site variant	Missense	Missense	Canonical splice site variant
?	?	?	?	Loss of aa from p.1 to p.137	?	?	?	?
Exon 9	Exon 9	Exon 9	Exon 9	Exon 10	Exon 11	Exon 12	Exon 12	Exon 14
Male	Male	Female	Male	Female	Female	Female	Male	Male
8 yo	5 yo	15 yo	6.10 yo	2.5 yo	13 yo	6 yo	14 yo	12 mo
Severe growth retardation	None	Short stature, underweight	Short stature, underweight, delayed bone age	Short stature, underweight	Short stature, underweight	Short stature, underweight	Short stature, underweight	50th percentile
Sat at 12 mo, Walked at 3 yo	N/A	Sat at 10 mo, Walked at 30 mo	Walked at 4 yo	Cannot sit or stand unsupported	Sat at 10 mo, Walked at 16 mo	Sat at 10 mo, Walked at 27 mo	Walked at 20 mo	No finalized movements, postural difficulties
Only few words	Mild delay	Short sentences	First words at 4 yo	Only few words	No speech problems	First words at 14 mo	First words at 4.5 yo	N/A
Synophrys, hypertrichosis, down-slanting palpebral fissures, bulnous nose	Low set hairline, thick eyebrows, unilateral partial ptosis, two rows of teeth	Broad nasal bridge, short upturned nose, arched eyebrows	Sparse scalp hair, pear-shaped nose, long flat philtrum, thin upper lip	Plagiocephaly, flat face, deep nasal bridge, retrognathia, low set ears	None	N/A	Bilateral epicanthus, small mouth, thin upper lip	None
Severe	No	Mild	Severe	N/A	Yes	N/A	Yes	N/A
Hypotonia, hyperactive, friendly behaviour	None	Hypotonia	N/A	N/A	Hypotonia, ADHD	Hypotonia, autistic traits, self-injuring, tantrums	Learning difficulties	Hypotonia, stereotyped movements
No	N/A	Febrile	N/A	N/A	No	No	N/A	N/A
N/A	N/A	Sleep apnea	N/A	N/A	Yes	N/A	N/A	Cycle disturbances
N/A	N/A	N/A	N/A	N/A	G-tube, constipation	No	N/A	N/A
N/A	N/A	N/A	N/A	N/A	N/A	N/A	N/A	Normal
Reduced anterior pituitary gland, delayed myelination	N/A	Volumetric loss in cerebral white matter around posterior horn of lateral ventriculum	Reduced size anterior pituitary gland	Normal	Normal	N/A	N/A	Mildly delayed myelination and mega cisterna magna
	Hyperlaxity of the joints		Diagnosed with TRPS		Scoliosis			

Suppl. table S1
