# Supplementary material for: Comparing Two Neurodevelopmental Disorders Linked to CK2: Okur-Chung Neurodevelopmental Syndrome and Poirier-Bienvenu Neurodevelopmental Syndrome—Two Sides of the Same Coin?
Source: Front Mol Biosci. 2022 May 26;9:850559. doi: 10.3389/fmolb.2022.850559 (PMC9182197; doi:10.3389/fmolb.2022.850559)
Supplement: Supplementary file 2 [file Table2.pdf]

Protein structure	**N-term**		**Autophosphorylation sites**								
	Patient G7	Patient G20	Patient F5	Patient A3	Patient G4	Patient G3	Patient G5	Patient E2	Patient G11	Patient G8	Patient G14
Publication	Ernst et al., Patient 7	Ernst et al., Patient 20	Yang et al., Patient 5	Li et al., Patient 3	Ernst et al., Patient 4	Ernst et al., Patient 3	Ernst et al., Patient 5	Poirier et al., Patient 2	Ernst et al., Patient 11	Ernst et al., Patient 8	Ernst et al., Patient 14
Amino acid change	p.(M1?)	p.(M1?)	p.(M1?)	p.R86C / **p.(E5*)**	p.(W9*)	p.(E20*)	p.(?)	p.(V25Mfs*13)	p.(G27D28dup)	p.(D32N)	p.(D32N)
Variant	c.1A>G	c.2T>A	c.3A>G	c.13G>T	c.27del	c.58G>T	c.73-2A>G	c.175+2T>G	c.78-83dup	c.94G>A	c.94G>A
Heritability	?	?	*De novo*	*De novo*	*De novo*	?	*De novo*	*De novo*	*De novo*	*De novo*	*De novo*
Classification	Start loss	Start loss	Start loss	Nonsense	Frameshift	Nonsense	Canonical splice site variant	Canonical splice site variant	In-frame duplication	Missense	Missense
Consequence prediction	New start codon at position p.52	New start codon at position p.52	New start codon at position p.52	No protein	No protein	No protein	?	No protein	No protein	?	?
Position	Exon 2	Exon 2	Exon 2	Exon 2	Exon 2	Exon 2	Intron 2	Intron 3	Exon 3	Exon 3	Exon 3
Sex	Male	Female	Female	Female	Male	Male	Male	Male	Male	Male	Male
Age at last follow-up	6 yo	13 yo	4.2 yo	17 mo	12 yo	36 yo	9 yo	10 yo	21 mo	12 yo	31 yo
Growth problems	Microcephaly	N/A	Underweight	Short stature	Failure to thrive	N/A	N/A	N/A	N/A	N/A	N/A
Motor development	Walked at 23 mo	Walked at 19 mo, poor coordination	Unsteady upright head	Sat at 1yo, cannot walk without help	Walked but stopped at 9 yo	Walked at 17 mo	Walked at 23 mo, slight dysarthria, incoordination	Crawled at 10 mo, walked at 18 mo	Walked at 15 mo	Walked at 2-3 yo	Walked at 2 yo
Speech development	Spoke at 28 mo	Spoke at 3.5 yo	N/A	N/A	Single words	Delayed	Spoke at 2 yo	N/A	Spoke at 21 mo	25 words at 5 yo	Few words
Dysmorphic features	Wide-based gait, mild hirsutism	N/A	N/A	N/A	N/A	N/A	N/A	N/A	N/A	Mild	Mild, wide-based ataxic gait
ID	N/A	Mild	Profound	Moderate	Profound	No	N/A	N/A	Mild	Moderate	Moderate
Neurological and Behavioural problems	Hypotonia	Hypotonia, autistic features, ADHD	N/A	N/A	Hypotonia	Autistic features, learning disability	Hypotonia	N/A	None	Hypotonia	None
Seizures	None	Present from 1 to 5 yo; polytherapy	Tonic-clonic, resolved with polytherapy	GTCS from 2 mo	Refractory myoclonic since 4 mo	GTCS from 1 to 10 yo	Febrile, then afebrile GTCS	Refractory myoclonic since 18 mo	Absences and GTCS since 5 mo; polytherapy	Febrile seizure at 1.5 mo	Refractory abscences and tonic spasms since 2 yo
Eating/Sleeping problems				N/A	G-tube	GERD	N/A	N/A	N/A	N/A	N/A
EEG	N/A	N/A	Sharper waves in the left area	Normal	N/A	N/A	Electrical status epilepticus during slow-wave sleep	Spike and polyspike waves gischarges and a slow background	N/A	N/A	N/A
MRI	T2 hypersensitivity and restricted diffusion of pontine central tegmental tracts	N/A	Abnomal signal shadow in temporal, occipital parietal lobe, hippocampus, corpus callosum	Poor myelination	N/A	N/A	N/A	Normal, but white hypersignals were observed	Two possible germinolytic cysts	Diffuse abnormal signal in cerebral white matter, low volume ventral pons	N/A
Others					Hearing loss						

KEN Box					D box		Acidic loop (AA 54-64)	Topoisomerase II i				
Patient G25	Patient G10	Patient G12	Patient B	Patient G6	Patient D	Patient G15	Patient G21	Patient G17	Patient G22	Patient A3	Patient A5	Patient G18
Ernst et al., Patient 25	Ernst et al., Patient 10	Ernst et al., Patient 12	Sakaguchi et al., Patient 1	Ernst et al., Patient 6	Selvam et al., Patient 1	Ernst et al., Patient 15	Ernst et al., Patient 21	Ernst et al., Patient 17	Ernst et al., Patient 22	Li et al., Patient 3	Li et al., Patient 5	Ernst et al., Patient 18
p.(D32N)	p.(F34S)	p.(N35K)	p.(T37Yfs)	p.(Q42*)	p.(R47*)	p.(R47*)	p(E61*)	p.(E77K)	p.(R86C)	**p.(R86C)/** p.G5*	p.(I88Ifs*46)	p.(M97I)
c.94G>A	c.101T>C	c.105T>A	c.108dup	c.124C>T	c.139C>T	c.139C>T	c.181G>T	c.229G>A	c.256C>T	c.256C>T	c.264delC	c.291G>A
De novo	*De novo*	*De novo*	*De novo*	*De novo*	*De novo*	*De novo*	*De novo*	*De novo*	*De novo*	*De novo*	*De novo*	*De novo*
Missense	Missense	Missense	Frameshift	Nonsense	Nonsense	Nonsense	Nonsense	Missense	Missense	Missense	Frameshift	Missense
?	?	?	No protein	No protein	No protein	No protein	No protein	?	?	?	No protein	?
Exon 3	Exon 3	Exon 3	Exon 3	Exon 3	Exon 3	Exon 3	Exon 4	Exon 4	Exon 4	Exon 4	Exon 4	Exon 4
Male	Female	Male	Male	Female	Male	Female	Male	Male	Female	Female	Male	Male
12 yo	17 yo	19 mo	21 mo	18 yo	17 yo	8 yo	11 yo	6 yo	3.5 yo	17 mo	1.8 yo	12 yo
N/A	N/A	Macrocephaly	Normal	N/A	Short stature until GH therapy	N/A	Macrocephaly, failure to thrive	Failure to thrive	N/A	Short stature	Short stature	N/A
Walked at 2 yo	Walked at 15 mo	Cannot sit unsupported	Sat at 8 mo, walked at 17 mo	Fine motor delay that improved	Walked at 18 mo	Walked at 15 mo, fine motor delay, poor coordination	Delayed walking	Walked at 21 mo	Walked at 13 mo	Sat at 1 yo, cannot walk without help	Normal	Walked at 17 mo, coordination problems
Delayed, speech dyspraxia	Delayed	Cannot babble	Meaningful words at 18 mo	N/A	Delayed	Simple sentences	Delayed	Spoke at 21 mo	Spoke at 8-9 mo	N/A	Normal	Spoke at 3 yo
Upslanting palpebral fissures, cleft soft palate, bifid uvula, asymmetry in limb creases	Mild	Mild, wide-based ataxic gait	None	N/A	Mild prognathism	N/A	Strabismus, nystagmus	Mild, cleft palate	N/A	N/A	N/A	Mild
Moderate	Mild	Moderate	N/A	No	Yes	Mild	Mild	No	No	Moderate	No	Mild
Hypotonia, anxiety, autistic features	Autistic features	None	N/A	Learning disability	Hypotonia, learning disability, comprehension deficit	Autistic features	Hypotonia, autistic features	Hypotonia, learning disability	None	N/A	N/A	N/A
Absences since 7 yo; polytherapy	None	None	Daily complex partial seizures since 10 mo; polytherapy	Febrile seizures with focal onset since 4 mo	Myotonic-atonic since 11 mo; polytherapy	Drop attacks since 2 yo; polytherapy	GTCS since 2 mo; polytherapy	None	Myoclonic absences since 14 mo	GTCS from 2 mo	GTCS since 6 mo	Refractory focal onset GTCS since 4 mo
N/A	Sleep problems	N/A	N/A	N/A	Swallowing difficulties	N/A	GI issues	N/A	N/A	N/A	N/A	N/A
N/A	N/A	N/A	Myoclonus of cortical origin with organized background activity	N/A	Bi-frontal spike-wave activity	N/A	N/A	N/A	N/A	Normal	Normal	N/A
N/A	Paraventricular gliosis	N/A	Normal	N/A	Normal	N/A	N/A	N/A	N/A	Poor myelination	Normal	N/A
					Mutation of FKRP							

				Zinc-finger domain (AA 105-146)							
				Cys 109		Cys 114			Cys 137		
nteraction domain					p53 interaction domain						
Patient F4	Patient E1	Patient G1	Patient G2	Patient G23	Patient A7	Patient A8	Patient A9	Patient G19	Patient G13	Patient A4	Patient A6
Yang et al., Patient 4	Poirier et al., Patient 1	Ernst et al., Patient 1	Ernst et al., Patient 2	Ernst et al., Patient 23	Li et al., 2019 Patient 7	Li et al., Patient 8	Li et al., Patient 9	Ernst et al., Patient 19	Ernst et al., Patient 13	Li et al., Patient 4	Li et al., Patient 6
p.(?)	p.(L98Afs*11)	p.(Y101*)	p.(Y101*)	p.(F106V)	p.(R111P)	p.(R111P)	p.(?)	p.(M132fs)	p.(C137R)	p.(C137G)	p.(C137F)
c.292-2A>T	c.367+2T>C		c.303C>A	c.316T>G	c.332G>C	c.332G>C	c.368-2A>G	c.394_404del	c.409T>C	c.409T>G	c.410G>T
De novo	*De novo*	*De novo*	*De novo*	*De novo*	*De novo*	*De novo*	*De novo*	*De novo*	*De novo*	*De novo*	*De novo*
Canonical splice site variant	Canonical splice site variant	Nonsense	Nonsense	Missense	Missense	Missense	Canonical splice site variant	Frameshift	Missense	Missense	Missense
?	?	No protein	No protein	?	?	?	?	No protein	?	?	?
Intron 4	Intron 5	Exon 5	Exon 5	Exon 5	Exon 5	Exon 5	Intron 5	Exon 6	Exon 6	Exon 6	Exon 6
Male	Male	Male	Male	Male	Male	Female	Female	Female	Male	Female	Female
1.2 yo	10 yo	6 yo	6 yo	4.5 yo	2 yo	1.3 yo	6 mo	15 yo	9 yo	14 mo	2 yo
Microcephaly, short stature	N/A	Microcephaly, failure to thrive	Microcephaly, failure to thrive	Macrocephaly	Short stature, underweight	Microcephaly, short stature	Slightly underweight	Macrocephaly, failure to thrive, delayed bone age	N/A	Short stature	Underweight
Unstaedy upright head at 8 mo	Crawled at 11 mo, walked at 24 mo	Walked at 5 yo	Walked at 6 yo	Walked at 17 mo	Still cannot walk without help	Sits at 7 mo; cannot walk without help	Rolled over at 4 mo, still cannot walk	Walked at 17 mo	N/A	Normal	Still cannot walk without help
Delayed	N/A	Nonverbal	Nonverbal	Speaks 30 words	Only several words	Cannot speak mom and dad	N/A	Spoke at 3 yo	Spoke at 2 yo	Normal	Only several words
N/A	Mandibular prognathism, mouth in fixed O position, tongue protrusion, bushy eyebrows, flat philtrum	Mild, wide-based gait, intermittent ptosis	Mild, wide-based gait, intermittent ptosis	Mild, wide spacing of teeth	N/A	N/A	N/A	Mild	Mild	N/A	N/A
Moderate	N/A	Profound	Profound	Mild	Mild	Mild	Mild	Mild	N/A	None	Mild
N/A	Hypotonia, nonspecific autistic traits, severe attention and learning difficulties	Hypotonia	Hypotonia	Hypotonia	N/A	N/A	N/A	Significant behavioural concerns	Autistic features	N/A	N/A
Tonic clonic; polytherapy	N/A	Myoclonic atonic since 6 mo; polytherapy	Myoclonic atonic since 10 mo; polytherapy	Febrile seizures since 3-4 mo	GTCS since 5 mo	GTCS since 5 mo	GTCS since 3.5 mo	GTCS at 3 and 5 yo	Focal onset GTCS since 6 mo; therapy until 6 yo	GTCS since 2 mo	GTCS since 6 mo
N/A	N/A	N/A	N/A	N/A	N/A	N/A	N/A	N/A	N/A	N/A	N/A
Sharp slow waves in temporal regions	Normal	N/A	N/A	N/A	Normal	Normal	Normal	N/A	N/A	Epileptiform discharge in temporal regions	Sharp waves in frontal, central, middle temporal and middle line regions
Epidural space widened and small subdural effusion	Normal	N/A	N/A	N/A	Normal	Normal	Normal	N/A	N/A	Normal	Normal
		Congenital heart disease		Mild laxity of finger joints				Mild scoliosis, GJB2 mutation			

Cys 140						Positive regulatory region (AA 187-193)				C-term
Patient F1	Patient C2	Patient F2	Patient F3	Patient C1	Patient G9	Patient G24	Patient G16	Patient F6	Patient A1	Patient A2
Yang et al., Patient 1	Nakashima et al., Patient 4	Yang et al., Patient 2	Yang et al., Patient 3	Nakashima et al., Patient 3	Ernst et al., Patient 9	Ernst et al., Patient 24	Ernst et al., Patient 16	Yang et al., Patient 6	Li et al., Patient 1	Li et al., Patient 2
p.(C137F)	p.(H165R)	p.(H165R)	p.(L167Sfs*60)	p.(P179Yfs*49)	p.(N181fs)	p.(?)	p.(?)	p.(?)	p.(L187R)	p.(F207Ffs*39)
c.410G>T	c.494A>G	c.494A>G	c.499delC	c.533_534insGT	c.542del	c.557+1G>A	c.558-2A>G	c.558-3T>G	C.560T>G	c.620_621insC
De novo	*De novo*	*De novo*	*De novo*	*De novo*	*De novo*	*De novo*	*De novo*	*De novo*	*De novo*	*De novo*
Missense	Missense	Missense	Missense	Frameshift	Frameshift	Canonical splice site variant	Canonical splice site variant	Canonical splice site variant	Missense	Frameshift
?	?	?	No protein	No protein	No protein	?	?	Exon skipping and new exon splicing enhancer	?	?
Exon 6	Exon 6	Exon 6	Exon 6	Exon 6	Exon 6	Intron 6	Intron 6	Intron 6	Exon 7	Exon 7
Female	Male	Male	Male	Female	Male	Male	Female	Male	Male	Female
4.2 yo	7 yo	3 yo	3.2 yo	15 yo	26 yo	22 yo	11 yo	7 mo	6.2 yo	3.5 yo
Underweight	Microcephaly, short stature, underweight	Short stature, underweight	Short stature, underweight	Microcephaly, short stature, underweight	Short stature	N/A	Failure to thrive	Normal	Microcephaly, short stature, underweight	Short stature, underweight
Could not sit at 7 mo	N/A	Unsteady upright head	Walks and runs alone	N/A	Walked at 7 yo, but now only with help	Walked at 18 mo	Motor delays, lack of balance	Cannot turn or sit alone	Sat at 1.5 yo, still cannot walk	Walked at 16.5 mo
Monosyllabic	N/A	Monosyllabic	Non verbal	N/A	Nonverbal, ataxia	Spoke at 2.5 yo	Spoke at 2 yo	Monosyllabic	N/A	Says five to six words
N/A	Inverted mouth space	N/A	N/A	None	Mild	N/A	N/A	N/A	N/A	N/A
Profound	Profound	Profound	Profound	Profound	Severe	Severe	Mild	Profound	Profound	Mild
Hyperactive, concentration difficulties	Dystonia	N/A	Cannot understand instructions	N/A	Hypotonia, autistic features	Cognitive deficits	No	N/A	N/A	N/A
Tonic-clonic since 5 mo	Focal seizures since 3 days	Myoclonic; polytherapy	Febrile	Facial clonic since 2 mo	Refractory GTCS, myoclonic and atypical absences since 10 mo	Refractory GTCS, myoclonic and absences since 2.5 mo	Focal onset GTCS, drop attacks; polytherapy	Tonic-clonic; polytherapy	Myoclonus since 1 yo, 2 GTCS	GTCS since 5 mo
N/A	GERD	N/A	N/A	N/A	N/A	N/A	N/A	N/A	N/A	N/A
Normal	N/A	More sharp waves in central, apical and midline areas	Normal	N/A	N/A	N/A	N/A	Sharp, spike and slow waves in frontal, central and temporal area	Diffuse and multiple spike-slow, slow wave discharging	Slow background and occasionally atypical spikes
Normal	Mega cisterna magna	Normal	Normal	Cerebellar atrophy	N/A	N/A	N/A	Normal	Normal	Slightly widen subarachnoid space
					Partial GH deficiency		Low bone density			

suppl. table S2
